# Impact of Compression on the Textural and Structural Properties of CPO-27(Ni)

**DOI:** 10.3390/molecules28196753

**Published:** 2023-09-22

**Authors:** Gabriel Trierweiler Gonçalves, Laure Michelin, Ludovic Josien, Jean-Louis Paillaud, Gérald Chaplais

**Affiliations:** 1 Université de Haute-Alsace, CNRS, Institut de Science des Matériaux de Mulhouse (IS2M), UMR 7361, Axe Matériaux à Porosité Contrôlée (MPC), F-68100 Mulhouse, France; 2 Université de Strasbourg, F-67000 Strasbourg, France

**Keywords:** MOF, CPO-27-Ni, shaping, compression, pelletization, adsorption, X-ray diffraction, optimization

## Abstract

The employment of metal-organic frameworks in powder form is undesirable from an industrial perspective due to process and safety issues. This work is devoted to evaluating the impact of compression on the textural and structural properties of CPO-27(Ni). For this purpose, CPO-27(Ni) was synthesized under hydrosolvothermal conditions and characterized. Then, the resulting powder was compressed into binderless pellets using variable compression forces ranging from 5–90 kN (37–678 MPa) and characterized by means of nitrogen adsorption/desorption, thermogravimetric analysis and powder X-ray diffraction to evaluate textural, thermal and structural changes. Both textural and structural properties decreased with increasing compression force. Thermal stability was impacted in pellets compressed at forces over 70 kN. CPO-27(Ni) pelletized at 5, 8 and 10 kN, and retained more than 94% of its initial textural properties, while a loss of about one-third of the textural property was observed for the two most compressed samples (70 and 90 kN) compared to the starting powder.

## 1. Introduction

Metal-organic frameworks (MOFs) have been extensively explored since the 90s, and thousands of new structures have been synthesized and reported over the years. These crystalline porous materials usually display thermal stability, high surface areas, high porosity with uniform pore size distribution, which can be tuned according to specific purposes, and offer the possibility to functionalize the framework. A large panel of topologies can be obtained thanks to the combination of different metals (e.g., zinc, copper, cobalt, nickel, magnesium, aluminum, indium, chromium, scandium, uranium, etc.) with different organic linkers (e.g., carboxylates, phenolates, phosphonates, imidazolates, amines, etc.) [1,2,3].

In this context, the Coordination Polymer of Oslo 27—CPO-27—(also known as MOF-74) is a metal-organic framework that assembles divalent metal cations (e.g., Ni^2+^, Co^2+^, Zn^2+^ and Mg^2+^) to the organic linker 2,5-dihydroxyterephthalate (dhtp) via the oxygen atoms of both the carboxylate and phenolate groups [4,5,6,7]. Regardless of the metal present in the composition, CPO-27 exhibits a honeycomb-like structure with a pore opening of around 11 Å. Each M^2+^ is also coordinated to an oxygen atom from an adsorbed water molecule, which can be desorbed upon heating, generating an unsaturated metal site [8,9], with a high potential for σ and π bond interactions [10,11]. Thus, this material can play a role in hydrogen storage [4,12], CO_2_ adsorption [13,14,15], CH_4_ capture [16], ammonia adsorption [17], xylene isomers separation [18], CH_4_/CO_2_ separation [19,20], CO_2_/N_2_ separation [21], ethane/ethylene separation [22], propane/propene separation [22,23], a ppb-level CO gas sensor [24,25], a catalyst for aldehyde cyanosilylation and styrene oxidation [26], a catalyst for ammonia synthesis [27], a catalyst for CO oxidation [28] and NO delivery for medical device applications [29].

From an industrial scale application perspective, powder materials are best avoided as they are generally not suitable for the process. In a reactor, for instance, powders are likely to agglomerate and consequently cause clogging, which has an impact on the system pressure. In addition, light powders are usually hazardous as the dust can be quickly inhaled or come into contact with the eyes, causing safety issues for the operator. These problems associated with the handling of powder materials are usually solved in the industry through the shaping process [30].

The shaping process (e.g., pelletization, extrusion and granulation) is a process in which the powder takes on a well-defined shape under the effect of stimuli (e.g., compression). Consequently, the material becomes significantly more manipulable and usable than the material in powder form. The shaped material must, however, be mechanically resistant to the process in which it is used and, finally, it must retain the intrinsic physico-chemical properties of the initial source [30,31].

Despite the density of information found in the literature concerning the syntheses approaches and the resulting physical-chemical properties, there is a lack of information regarding the impact of shaping on the material properties. It is, however, worth highlighting the importance of shaping for the viability of industrial applications [32].

Therefore, this present work aims to assess the impact on the textural, thermal and structural properties of CPO-27(Ni) upon compression. To accomplish it, the synthesized powder was compressed using a wide range of compression forces and nitrogen adsorption measurements, and thermogravimetric analyses and X-ray diffraction experiments were carried out on the powder and compressed samples. The data obtained from the mentioned analyses of the starting material and compressed samples were further compared and discussed.

## 2. Results and Discussions

### 2.1. Starting Material Characterization

**Figure 1:**
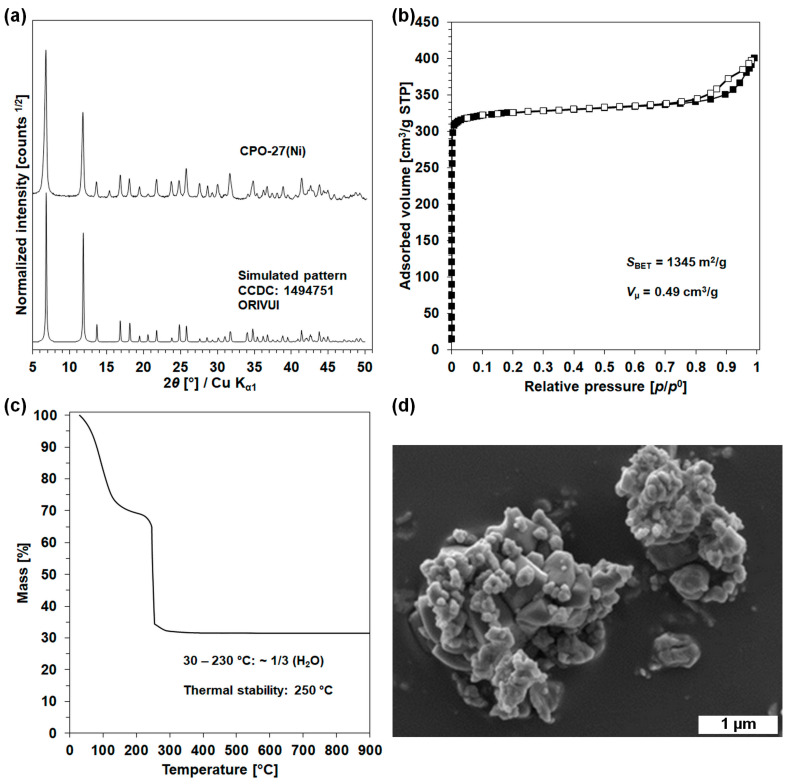
Characterization of starting material with (**a**) X-ray diffraction pattern, (**b**) N_2_ adsorption/desorption isotherm at −196.15 °C, (**c**) TGA profile and (**d**) SEM image of CPO-27(Ni).

The powder X-ray diffraction (PXRD) pattern, the isotherm of nitrogen adsorption/desorption at −196.15 °C, thermogravimetric analysis (TGA) profile and scanning electron microscopic (SEM) image of CPO-27(Ni) starting material are grouped in Figure 1.

The powder X-ray diffraction pattern of CPO-27(Ni) is plotted in Figure 1a. The diffraction pattern was identified to its corresponding compound (CCDC 1494751, Refcode ORIVUI [33]) and it was further indexed using the STOE WinX^POW^ software. The sample had a honeycomb-like structure and the refined unit cell parameter in the space group *R*-3 was found to be *a* = 25.943(2) Å and *c* = 6.694(2) Å for CPO-27(Ni), which is in good agreement with the literature results (*a* = 25.9783(7) Å and *c* = 6.6883(2) Å) [4].

The nitrogen adsorption/desorption isotherm measured at −196.15 °C is shown in Figure 1b. CPO-27(Ni) has a Type I isotherm according to IUPAC classification, which is characteristic of microporous adsorbents [34]. In addition, a hysteresis loop can be observed between *p*/*p*^0^ = 0.8–0.99, which is attributed to the intergranular porosity along with the condensation phenomena. The surface area (*S*_BET_) and micropore volume (*V*_μ_) of CPO-27(Ni) are, respectively, 1345 m^2^/g and 0.49 cm^3^/g. These textural properties are in good agreement with those expected according to the previous findings (1218 m^2^/g and 0.47 cm^3^/g [19]), while even slightly higher than them.

The TGA profile of the starting powder CPO-27(Ni), recorded several days after its synthesis, is shown in Figure 1c. It can be seen that CPO-27(Ni) contains significant amounts of water, as the weight loss between 30 and 230 °C is 32%. This is consistent with the results reported by Rosnes et al. [35], who stated that all of the CPO-27(M) samples (M = Zn, Co, Ni, Mg, Mn, Cu) that they synthesized displayed a high affinity for water, with a water uptake capacity of about 30 wt.%. The experimental and theoretical weight losses between 200 and 800 °C of CPO-27(Ni) are in good agreement (53 and 52 wt.%, respectively, considering the weight loss, with NiO formation at the end of the analysis). Moreover, the TG curve reveals an abrupt collapse of the structure at 250 °C, which is in good agreement with the literature, giving a temperature of ~240–250 °C) [4].

The SEM image of CPO-27(Ni) powder is shown in Figure 1d. CPO-27(Ni) presents a poorly defined morphology, in which the particles with a heterogeneous size ranging from 150–400 nm are present as aggregates measuring 4 μm on average.

### 2.2. Pelletization

**Figure 2:**
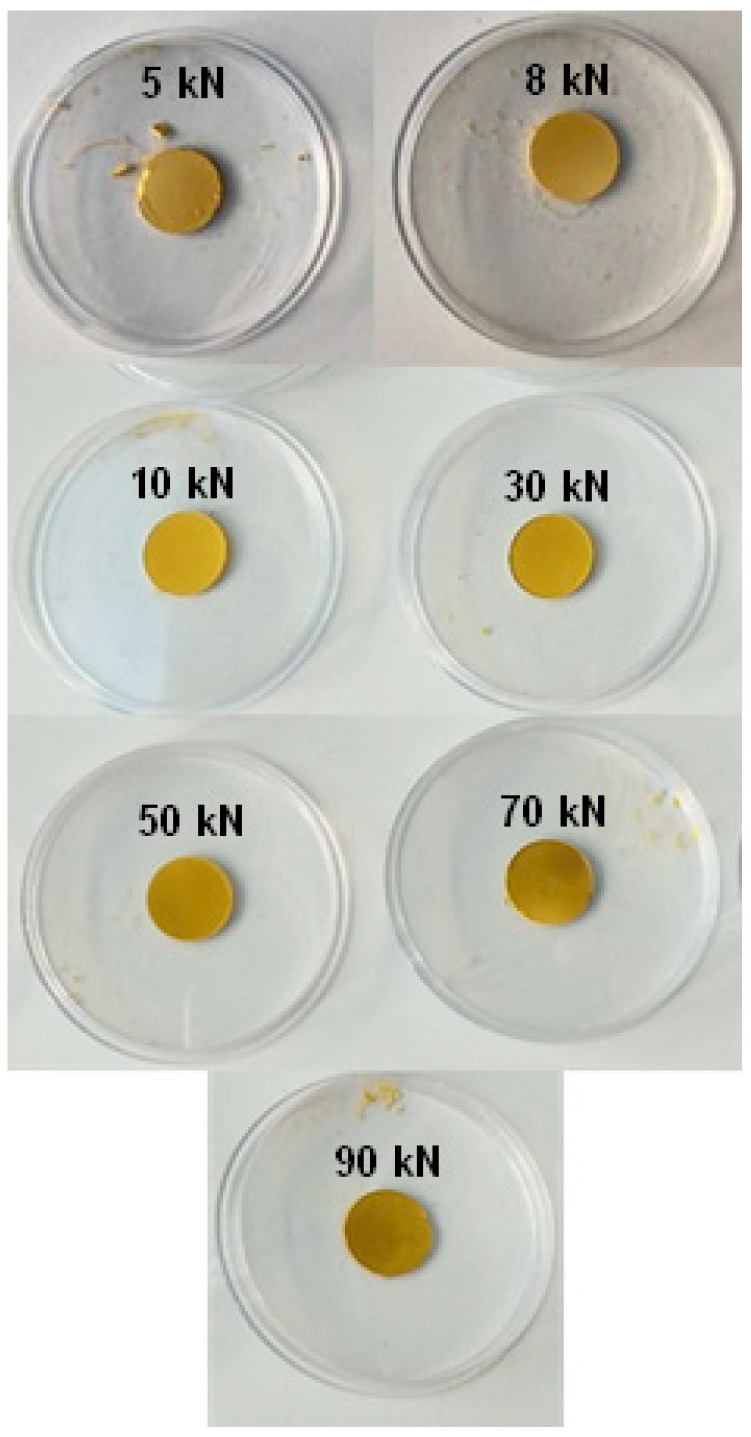
Color evolution of the compressed CPO-27(Ni) pellets at different compression forces.

The pelletized samples are shown in Figure 2. It is worth mentioning that the pellets were prepared without the use of any binder. In terms of visual appearance, the three least compressed samples (CPO-27(Ni)-5, CPO-27(Ni)-8 and CPO-27(Ni)-10) retained the same color as the starting powder. At higher pressure, the pellets became darker as the compression force increased (from yellow to dark yellow).

The pelletization of CPO-27(Ni) resulted in hard pellets with a smooth surface for all applied compression forces with the exception of CPO-27(Ni)-5. The latter had a lower mechanical resistance than all other compressed pellets. In fact, CPO-27(Ni)-5 crumbled into small pellet pieces upon any impact and also when cut in half with a razor blade. In contrast, all the other pellets did not crumble when cut in half with the razor blade. In fact, they give two equal half disks with a clean-cut section. It should be noted that the last two most compressed samples (CPO-27(Ni)-70 and CPO-27(Ni)-90) were particularly difficult to cut in half, as they presented a texture similar to that of a hard plastic (PMMA for instance). Finally, CPO-27(Ni) compressed at 8 and 10 kN showed almost identical textural properties.

### 2.3. Nitrogen Sorption Analyses

**Figure 3:**
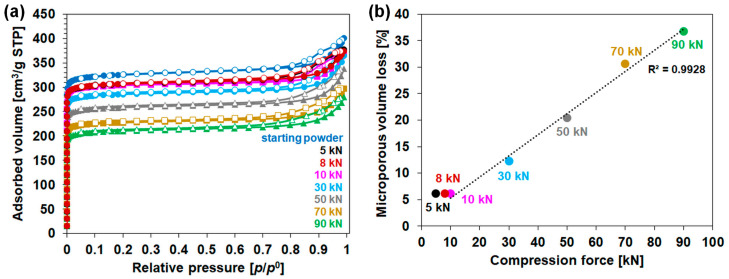
Textural properties of starting powder and pelletized CPO-27(Ni) with (**a**) nitrogen adsorption/desorption isotherms at −196.15 °C, and (**b**) evolution of micropore volume loss in function of compression force with dotted line representing a linear fit in the range 10–90 kN.

Nitrogen adsorption and desorption isotherms of the starting CPO-27(Ni) and compressed samples are shown in Figure 3, and the textural properties (surface area and micropore volumes) extracted from the isotherms are presented in Table 1. It is worth noting that the weight losses observed during degassing (30–35 wt.%) are similar to that measured (32 wt.%) through thermogravimetric analyses between room temperature and 230 °C. This weight loss is assigned to the removal of both chemisorbed and physisorbed water molecules (Table 2). These findings reveal that the degassing operating conditions (i.e., 12 h at 200 °C under a secondary vacuum) are suitable for dehydrating the powder and the pellets.

Figure 3 shows that the starting and pelletized CPO-27(Ni) samples display the same profile of adsorption isotherm. Moreover, the volume of nitrogen adsorbed on the material decreases with the increasing compression force. The micropore volume loss upon compression follows a linear trend for the compression forces in the range of 10 and 90 kN, as shown in Figure 3b. The BET surface area loss follows the same trend with almost the same values. In fact, more than 10% of the initial textural properties are lost upon compression using compression forces above 30 kN. This decrease reaches about one-third for samples compressed at 70 and 90 kN (Table 1). In contrast, the less-compressed samples (5, 8 and 10 kN) retain almost all integrity of textural properties with a surface area and micropore volume near those of the starting CPO-27(Ni), with only 6 wt.% loss. These results illustrate the negative impact of high compression force on the textural properties of CPO-27(Ni) pellets as partially evidenced by previous works [16,17].

### 2.4. Thermogravimetric Analyses

**Figure 4:**
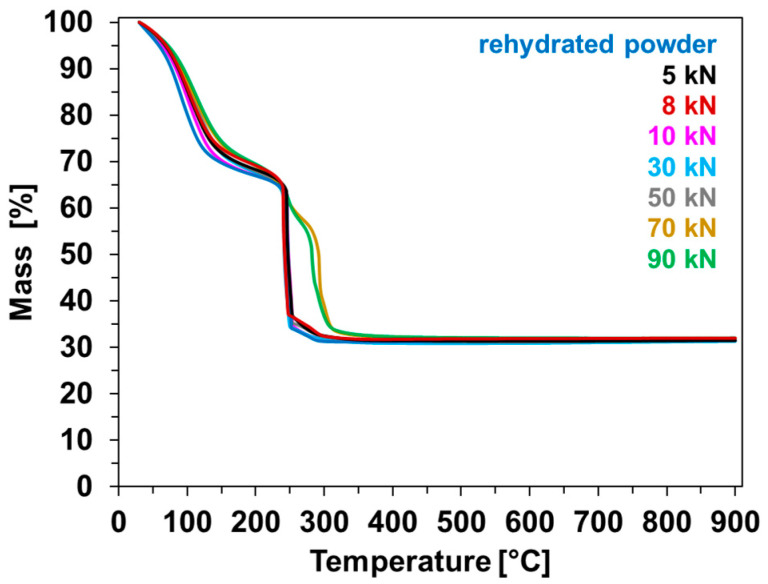
Thermogravimetric profiles of starting and pelletized CPO-27(Ni) samples.

The thermogravimetric profiles of the powder and compressed samples, carried out two weeks after nitrogen sorption analyses, are presented in Figure 4. Both powder and pelletized samples feature almost the same amount of water; i.e., 32–35 wt.% (weight losses measured between 30 and 230 °C). This similarity is also observed in the weight loss of the freshly synthesized CPO-27(Ni) powder, as indicated in Table 2 (Section 2.3). These results reveal that the two-week period after the nitrogen sorption analyses, during which the samples (half pellets and powder) were exposed to atmospheric moisture, is sufficient to restore them to their initial hydration state. The chemical formula for all of the samples is thus: Ni_2_(H_2_O)_2_(dhtp)•~7H_2_O. As previously mentioned, these weight losses are also of the same magnitude as those observed during the degassing step during nitrogen sorption. This also suggests that the majority of acid Lewis sites (i.e., coordinated Ni atoms) are unoccupied in the degassed samples.

Exploring the contrasting behaviors of CPO-27(Ni) pellets concerning water and nitrogen offers intriguing insights. In Section 2.3, it was determined that compression exerts a substantial influence on nitrogen adsorption capacities, particularly beyond 10 kN. On the other hand, the TG results show that the initial hydration rate of the degassed samples is recovered after two weeks of exposure to atmospheric air. These findings not only underscore the hydrophilic nature of CPO-27(Ni) but also suggest that limitations in gas or vapor accessibility due to compression forces are contingent on the adsorbate. This phenomenon is related to the kinetic diameter of the absorbent molecules, given that those of water and nitrogen are 2.641 and 3.64–3.80 Å, respectively [36].

Moreover, regarding the analysis of the TG curves, both the rehydrated powder and the compressed pellets within the range of 5–50 kN display the same profile as the starting powder, with the structural collapse centered at 250 °C. Surprisingly, the pellets CPO-27(Ni)-70 and CPO-27(Ni)-90 also start to collapse at 250 °C. However, the structure breakdown persists until about 300 °C, at which point the samples fully collapse. This delay in the complete structure collapse may suggest diffusion issues due to the intense sample compression, even if it is difficult to see a large difference in compaction among the samples in view of the SEM results (Section 2.6).

### 2.5. Powder X-ray Diffraction Analyses

**Figure 5:**
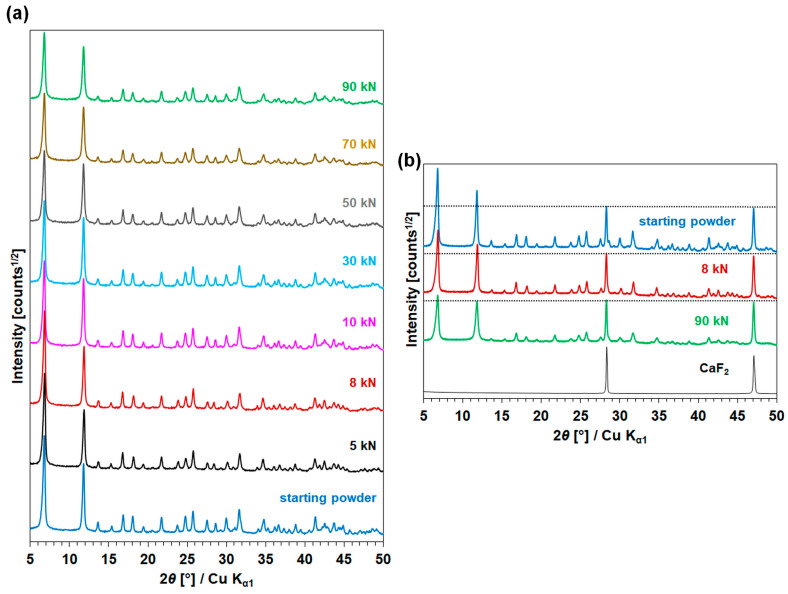
PXRD patterns of (**a**) starting powder and pellets compressed under increasing pressures, (**b**) starting powder, CPO-27(Ni)-8 and CPO-27(Ni)-90 mixed with 25 wt.% of CaF_2_ where dotted line denotes the intensity of the first CaF_2_ peak at ~28.5° in 2*θ*. For clarity, a *y*-axis offset is applied to each of the PXRD patterns.

The PXRD patterns of the starting CPO-27(Ni) and compressed samples are shown in Figure 5 and their refined unit-cell parameters are listed in Table 3.

Figure 5a regroups the patterns of the CPO-27(Ni) starting material and pellets following compression under various pressures. The patterns display similarities in their profiles and peak positions. Consequently, the pelletized samples, like the initial CPO-27(Ni) sample, can be confidently identified as pure CPO-27(Ni). This identification aligns well with the crystallographic data presented in the structure referenced as CCDC 1494751, with the Refcode ORIVUI [33]. However, it is notable that the compression process affects the crystallinity, as the diffraction peaks’ intensities decrease as a function of increased pressure. To eliminate any potential misinterpretation associated with fluctuations in the diffracting volume, a series of measurements were undertaken using an internal standard. In Figure 5b, the XRD powder patterns are normalized to the first CaF_2_ peak (at ~28.5° in 2*θ*), which serves as an internal standard. A decrease in the peaks’ relative intensity is also observed as a function of compression force increase. This phenomenon is all the more visible for the first two peaks, which are also the most intense, at 6.8 and 11.8° in 2*θ*, corresponding to the planes (010) and (200). This is attributed to the partial amorphization of CPO-27(Ni) upon compression stimuli. As shown in Section 2.3, this trend is concomitant with a decrease in textural properties emphasized in nitrogen sorption analyses.

Table 3 shows the refined unit-cell parameters in the space group *R*-3 for the starting CPO-27(Ni) and pelletized samples. It can be seen that the process of pelletization of CPO-27(Ni) did not impact the initial unit-cell parameters for the compressed pellets between 10 and 90 kN, even if the intensity changes of the diffraction peaks reveal a partial amorphization. This is evidenced by the absence of a peak shift between the starting material and the compressed samples. Nonetheless, the refined unit-cell parameters of the CPO-27(Ni)-5 and CPO-27(Ni)-8 samples are slightly affected by the impact of the compression. The possible reason(s) for such phenomena remain(s) unclear to this date, and a thorough investigation should be undertaken in order to shed light on this issue.

### 2.6. Scanning Electron Microscopy

**Figure 6:**
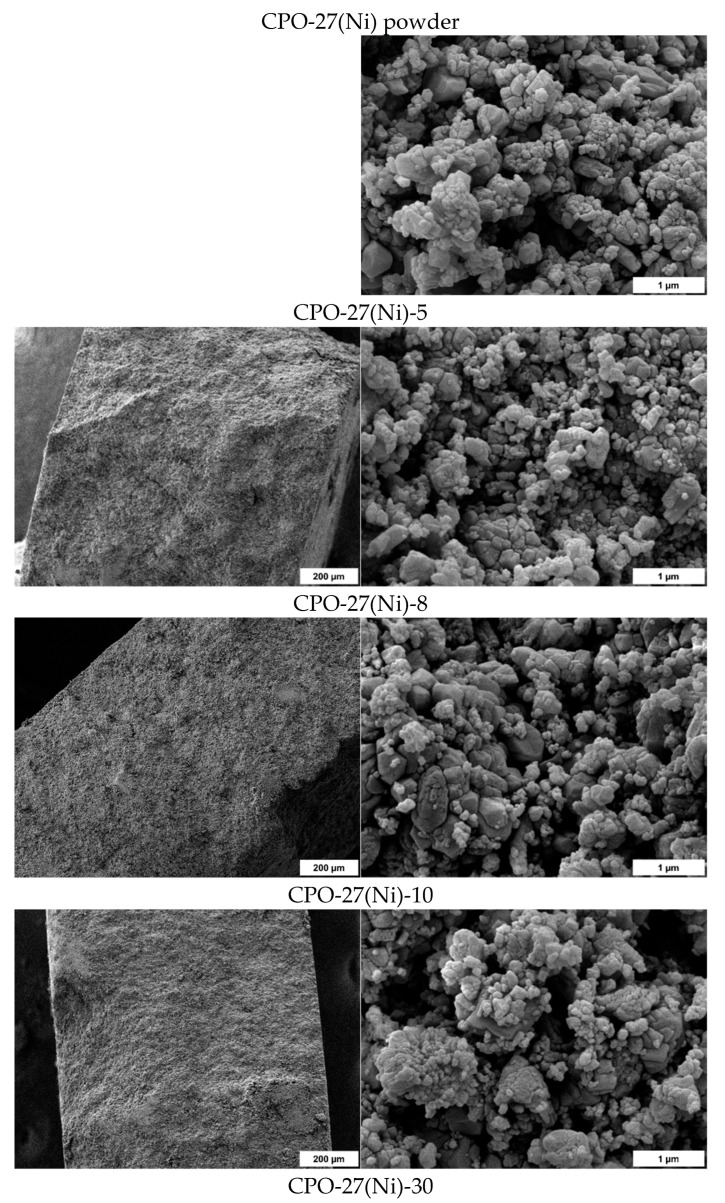

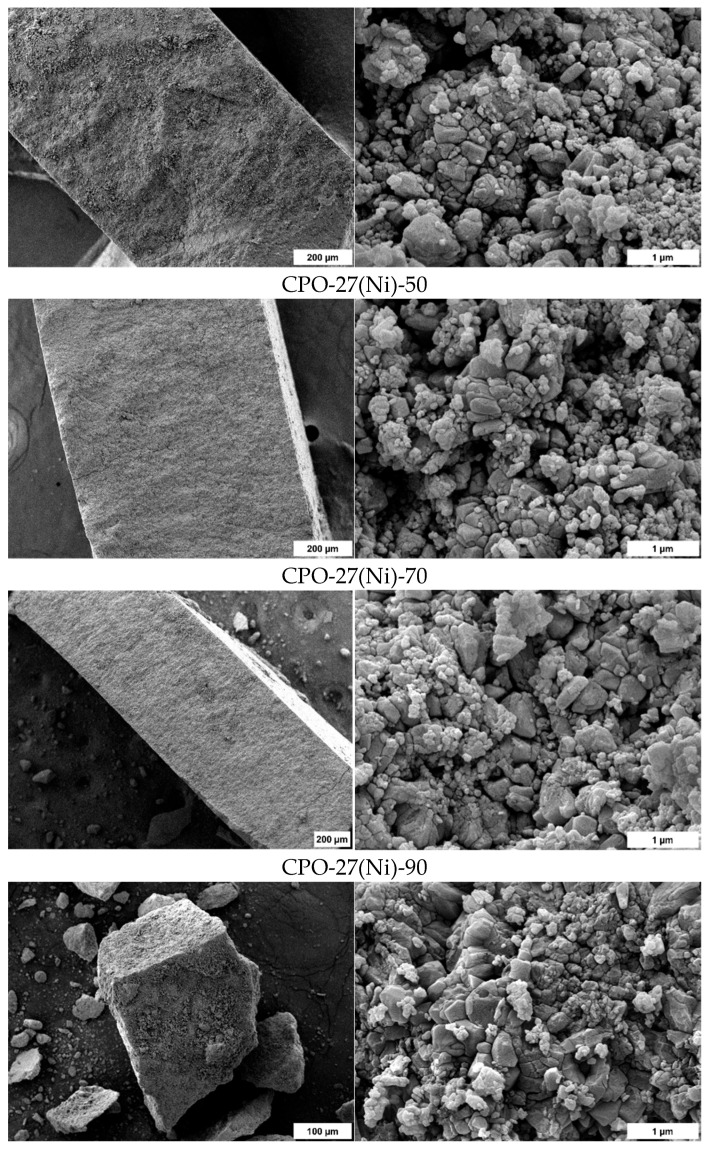
SEM images of CPO-27(Ni) compressed samples with a cross-sectional view of pellets with a low (**left images**) and high magnification (**right images**).

Figure 6 displays SEM images presenting a cross-sectional view of the compressed samples, excluding the powder sample and the CPO-27(Ni)-90 pellet due to the additional analyses required for the latter. The SEM images, captured at both low and high magnifications, reveal a distinctively well-defined cut surface in the samples, particularly pronounced in those compressed at higher pressures, such as CPO-27(Ni)-50 and CPO-27(Ni)-70. Regarding particle sizes, the application of elevated pressures in pellet fabrication did not lead to significant alterations. The images exhibit a bimodal particle distribution, with one group consisting of micrometric-sized particles and another featuring particles of a few hundred nanometers. This is consistent with the XRD results, where the full width at half maximum of the diffraction peaks does not significantly change (Section 2.5). Furthermore, it is notable that the particles within the pellets may exhibit a higher degree of compaction compared to those found in the powder. This phenomenon can be attributed to the settling of agglomerated particles from the powder due to the constraints imposed by the applied mechanical force during the compression process. This settling effect contributes to the increased density and more closely packed structure observed in the pelletized samples.

## 3. Materials and Methods

### 3.1. Materials

#### 3.1.1. Raw Materials

Nickel acetate tetrahydrate (Ni(CH_3_COO)_2_•4H_2_O, Fluka ≥ 99%), 2,5-dihydroxyterephthalic (H_4_dhtp, C_8_H_6_O_6_, Sigma Aldrich ≥ 98%), deionized water (H_2_O, 18 MΩ.cm^−1^), ethanol (EtOH, C_2_H_6_O, Carlo Erba ≥ 99.9%) and tetrahydrofuran (THF, C_4_H_8_O, Carlo Erba ≥ 99.9%) were used to prepare the CPO-27(Ni).

#### 3.1.2. Synthesis of CPO-27(Ni) Powder

CPO-27(Ni) (Ni_2_(dhtp)(H_2_O)_2_•8H_2_O) was synthesized following a procedure described in the literature [12] but adapted with a scale-up factor of 4. Ni(CH_3_COO)_2_•4H_2_O (5.2493 g, 21.10 mmol) was dissolved in 35.19 g of H_2_O (giving solution S1) in a 150-mL PTFE-lined stainless-steel autoclave, and H_4_dhtp (2.0902 g, 10.55 mmol) was separately dissolved in 31.32 g of THF (giving solution S2). S2 was then poured into S1 (autoclave filling level: 65%), yielding a greenish to yellowish suspension. The experimental molar composition was 1 Ni: 0.5 H_4_dhtp: 97 H_2_O: 21 THF. The final mixture was heated at 110 °C for 72 h. After cooling down, the suspension was filtered under a vacuum and the dark yellow solid was washed with 3 portions of 50 mL of H_2_O. The solid was then immersed in 325 mL of EtOH, dispersed for 3 min under sonication and then left static for 24 h. The solid was then filtered, washed with 3 portions of 50 mL of EtOH and finally dried at 80 °C for 2 h at room temperature overnight. Obtained mass: 2.8936 g. Yield: 88% (based on dried mass upon mass loss until 200 °C, determined by TGA). The synthesized sample was further used as a starting material to prepare pellets using different compression forces.

#### 3.1.3. Pelletization

An amount of 150 mg of CPO-27(Ni) was grinded and subsequently pressed at room temperature without the implementation of any additional vacuum. This pressing step was carried out using a Herzog TP40 (Herzog Maschinenfabrik, Osnabrück, Germany) equipped with a 13-mm pellet die set (International Crystal Laboratories). The pressing was conducted for a duration of 2 min, applying specific compression forces as follows: 5, 8, 10, 30, 50, 70 and 90 kN, which correspond to compression forces of 37.7, 60.3, 75.3, 226.0, 376.7, 527.4 and 678.1 MPa. The compressed CPO-27(Ni) samples were named as follows: CPO-27(Ni)-5, CPO-27(Ni)-8, CPO-27(Ni)-10, CPO-27(Ni)-30, CPO-27(Ni)-50, CPO-27(Ni)-70 and CPO-27(Ni)-90.

### 3.2. Characterization Techniques

#### 3.2.1. Nitrogen Sorption Measurements at −196.15 °C

Nitrogen adsorption/desorption analyses of the starting and compressed CPO-27(Ni) samples were performed at −196.15 °C using a Micromeritics ASAP 2420 (Micromeritics Instrument, Corp., Norcross, GA, USA). The samples (150 mg) were previously degassed under a vacuum at 200 °C for 12 h (the pelletized samples were cut in half using a razor blade in order to fit inside the tube). Textural properties information, such as the specific surface area (*S*_BET_) and microporous volume (*V*_μ_), were determined from the given isotherms. For each sample, *S*_BET_ was determined based on the criteria given in the literature [37,38], in the 0.0002 ≤ *p*/*p*^0^ ≤ 0.0160 range, while *V*_μ_ was determined according to the *t*-plot method (Harkins and Jura model).

#### 3.2.2. Powder X-ray Diffraction (PXRD)

The PXRD patterns of the starting powder and compressed CPO-27(Ni) samples after nitrogen adsorption/desorption analysis were recorded in transmission mode on a STOE STADI-P diffractometer (STOE & Cie GmbH, Darmstadt, Germany) equipped with a curved germanium (111) primary monochromator, and a linear position-sensitive detector (6° in 2*θ*) operating with Cu K_α1_ radiation (K_α1_ = 0.15406 nm). After grinding 20 mg, the samples were disposed of between two X-ray transparent cellulose acetate foils. The PXRD patterns were collected at room temperature in the 5° < 2*θ* < 50° range, at a step of 0.04° in 2*θ* and with a time of 25 s per step. The samples were analyzed by means of PXRD two weeks after the nitrogen adsorption/desorption analysis in order to allow sample rehydration.

The PXRD patterns were also recorded in Debye-Scherrer mode for the starting CPO-27(Ni), CPO-27(Ni)-8 and CPO-27(Ni)-90. The samples were mixed with an internal standard (CaF_2_). An amount of 20 mg (75 wt.%) of the sample and 6.67 mg (25 wt.%) of CaF_2_ were thoroughly mixed, ground and the resultant mixture was inserted into capillaries. The XRD patterns were collected at room temperature in the 5° < 2*θ* < 50° range, at a step of 0.1° in 2*θ* and with a time of 60 s by step.

#### 3.2.3. Thermogravimetric Analyses

Thermogravimetric analyses (TGA) of the powder and compressed samples were carried out using a TG Mettler Toledo STARe (Mettler Toledo, GmbH, Wien, Austria), under synthetic air flow, with a heating rate of 2 °C/min from 30 to 900 °C. The pellets were first crushed before being placed in alumina crucibles for analysis. The measurements were conducted two weeks after the nitrogen adsorption/desorption analyses, in order to make sample rehydration possible. The starting powder was analyzed several days after its preparation and also two weeks after the nitrogen adsorption/desorption analysis for comparison with the pellets.

#### 3.2.4. Scanning Electron Microscopy

Scanning electron microscopy images were recorded using a JSM-7900F (JEOL, Ltd., Tokyo, Japan) scanning electron microscope. Before analysis, the sample was coated with a fine carbon (in the case of the image for the starting powder in Section 2.1) or gold (in the case of the images presented in Section 2.6) layer using a SCD004 sputter coating system (BAL-TEC, LEICA MICROSYSTEMES SA) in order to improve the electrical conductivity.

## 4. Conclusions

CPO-27(Ni) was synthesized and binderless pellets were prepared in the range of 5 to 90 kN of compression force with a compression time of 2 min. The starting material and pelletized samples were characterized to evaluate the textural, thermal and structural impacts on CPO-27(Ni) upon compression. The less compressed pellet (5 kN) did not show good mechanical resistance compared to the other pellets. The pellets compressed at 5, 8 and 10 kN retained more than 94% of the initial textural properties, whereas the samples compressed at compression forces higher than 30 kN lost 10% and even around one-third of their textural properties, compared to the starting material, for the two most compressed samples (70 and 90 kN). The thermal behavior remained unchanged up to 50 kN. In addition, powder X-ray diffraction measurements revealed a loss of crystallinity upon compression. Despite this, the refined unit-cell parameters of pelletized samples in the range of 10–90 kN remained unchanged in comparison with the starting material, whereas those of the samples pelletized at 5 and 8 kN were slightly affected without deviating from the global trend. Therefore, the compression forces of 8 and 10 kN were proven to be the most suitable for producing a CPO-27(Ni) pellet with good texture and good mechanical resistance while almost maintaining the integrity of the textural and structural properties of the starting CPO-27(Ni) material. Finally, it is important to note that it is not necessary to add a binder to produce pellets with good mechanical and adsorption properties.

## Figures and Tables

**Table 1:** Textural properties of powder and pelletized CPO-27(Ni).

Sample	Pressure [MPa]	*S*_BET_ [m^2^/g]	*S*_BET_ Loss ^1^ [%]	*V*_μ_ [cm^3^/g]	*V*_μ_ Loss ^2^ [%]
CPO-27(Ni)	-	1345	-	0.49	-
CPO-27(Ni)-5	37.7	1266	6	0.46	6
CPO-27(Ni)-8	60.3	1267	6	0.46	6
CPO-27(Ni)-10	75.3	1258	6	0.46	6
CPO-27(Ni)-30	226.0	1182	12	0.43	12
CPO-27(Ni)-50	376.7	1068	21	0.39	20
CPO-27(Ni)-70	527.4	937	30	0.34	31
CPO-27(Ni)-90	678.1	865	36	0.31	37

^1^ *S*_BET_ loss compared to the departure material. ^2^ *V*_μ_ loss compared to the departure material.

**Table 2:** Weight losses observed during nitrogen sorption measurements and TG analyses.

Sample	Weight Loss by Degassing ^1^ [%]	Weight Loss by TGA ^2^ [%]
CPO-27(Ni)	30	32 ^3^/32 ^4^
CPO-27(Ni)-5	34	34
CPO-27(Ni)-8	34	33
CPO-27(Ni)-10	35	35
CPO-27(Ni)-30	35	35
CPO-27(Ni)-50	35	35
CPO-27(Ni)-70	34	34
CPO-27(Ni)-90	34	34

^1^ Weight loss resulting from N_2_ sorption degassing step at 200 °C for 12 h under secondary vacuum. ^2^ Weight loss issued from TG analysis under synthetic air at 2 °C/min between room temperature and 230 °C. ^3^ Weight loss issued from TG analysis between room temperature and 230 °C for freshly synthesized CPO-27(Ni) powder. ^4^ Weight loss issued from TG analysis between room temperature and 230 °C for degassed CPO-27(Ni) powder two weeks after nitrogen sorption analyses.

**Table 3:** Refined unit-cell parameters of powder and pelletized CPO-27(Ni) in the space group *R*-3.

Sample	Pressure [MPa]	*a* = *b* [Å]	*c* [Å]	Volume [Å^3^]
CPO-27(Ni)	-	25.943(2)	6.694(3)	3901.6(5)
CPO-27(Ni)-5	37.7	25.817(2)	6.761(4)	3883.1(3)
CPO-27(Ni)-8	60.3	25.812(3)	6.757(5)	3898.6(8)
CPO-27(Ni)-10	75.3	25.937(3)	6.702(6)	3904.5(8)
CPO-27(Ni)-30	226.0	25.930(2)	6.700(5)	3901.3(6)
CPO-27(Ni)-50	376.7	25.926(2)	6.702(4)	3901.4(5)
CPO-27(Ni)-70	527.4	25.932(2)	6.706(3)	3905.5(4)
CPO-27(Ni)-90	678.1	25.928(2)	6.707(3)	3904.8(4)

## Data Availability

Not applicable.

## Sample Availability

Samples of the compounds are not available from the authors.
